# Everybody's Page

**Published:** 1889-07-27

**Authors:** 


### Everybody's Page.

In 1789 the police of Paris were doing their best to prevent the sale of melons, oysters, ice, and other things, on the ground that they were unworthy to enter the human body.

A writer in the *Journal d'Hygiene* declares that the French are the most patient people on the face of the earth, and brings forward as evidence the patience with which they wait for hours till they can get a seat on an omnibus on going to or leaving the Exhibition.

Surgeon R. H. Firth, of the Army Medical Staff, has been making experiments with kola nut as a means of preventing fatigue and hunger. He is of opinion that it is useful, but only as an artificial stimulant to the nervous system, not as a substitute for food.

Our own General Medical Council is sometimes said to be too exacting in its reading of the demands of professional conduct, but it is nothing to the Medical Association of Alabama, which forbids its members to do contract practice —what we should call club work.

The phonograph has been used recently to record the sounds given by the heart and lungs under auscultation. This should be invaluable in consultation, as a true account of the patient's condition can thus be sent to a doctor at a distance ; and also the phonographic record may be used to compare the patient's present with his past condition, with an accuracy beyond that of the memory.

Some doctors, in their anxiety to explain the cause of flat-foot, have come to the conclusion that it is the normal foot that is automatically incorrect. Certainly all babies are flat-footed when they first begin to walk, and it is only by exercise and the development of the muscles of the legs that the arched instep comes into being and the sole of the foot is raised from the ground.

Many evil things have been laid at the door of competitive examination, but the worst is reported from China. The Chinese are inveterate gamblers, and they have taken to betting largely on examination results. It is said that learned tutors sell "tips" about their pupils to the public, and the book-makers and students, more shrewd than honest, who are doubtful as to their own chances, indulge in much ingenious hedging. It is to be hoped the custom will not spread to England.

It is very difficult to realise—what nevertheless is a fact—the existence of a trance other than cataleptic. Yet there have been known cases, especially in habitual inebriates, where the patients go about the world, sane to all appearance, but liabie to commit the most extraordinary acts, including theft and other crimes, of which in their ordinary condition they can recall nothing. They are not drunk, in the ordinary sense of the term, but the habit of drinking has produced a partial paralysis of brain-power.

The fierce light of the photographic camera has been brought to bear on the unwilling ghost, and specimens of "spirit photography" are being sold in the shops at two shillings each. They consist of a startled mortal looking up from his desk at something of vaguely human outline in the back-ground. Of course you must assume that this something really is a spirit, which, doubtless, some materialists will not grant, but if that be admitted, the portrait is as satisfactory as need be, though it must be confessed that beauty seems rather conspicuous by its absence in spirit-land.

Hypnotism has been good "copy" for novelists for some time. Now the artists are making their claim, and a Munich painter has produced a picture of a beautiful girl, asleep or entranced, which he has entitled "hypnotised."

Dr. Joseph Price, of Philadelphia, is a pronounced opponent of "Listerisms," as he calls antiseptic surgery. He says that it "works death by chemicals instead of dirt," and that cleanliness, water, and good surgery are the best of antiseptics.

Dr. G. F. Shurley, who has tried the hot-air treatment in cases of phthisis, reports on it unfavourably. In only one case had it any good effect; in the others it had to be stopped after a few days, as causing unendurable discomfort —often fever and nausea—to the patients.

A gentleman has discovered a method of preventing rabies in dogs. Give them a vegetarian diet, with unlimited cherries and strawberries. We should like to know if the same regimen acts as a prophylactic or cure for hydrophobia. If so we know quite a number of people who are willing to undergo the treatment.

"Wash not at all" is, says Dr. D. Mackintosh, the first commandment in the treatment of eczema. Washing keeps the skin dry, and favours the development of eczema by depriving it of the natural secretions on which its suppleness, softness, and beauty largely depends. But it is a terrible prescription to inflict on a bath-loving Briton.

The contagiousness of phthisis has been recognised in Germany by military command. An edict has gone forth from the War Minister that the chest of every soldier shall be examined once a month. If it does not measure enough, and does not expand with drill and athletic exercises, he is dismissed from the army as being predisposed to phthisis, and likely to infect his comrades.

Carriages appeared in England first under the reign of Elizabeth, and were fairly common by 1605. These were, however, private vehicles. But in 1634 a retired sea-captain, of the name of Baily, by way of exercising his horses during the winter, harnessed them to four carriages and sent them to one of the London thoroughfares with his servants, who were instructed to offer them to the public at a fixed tariff. The experiment was successful, and the hackney carriage became a recognised institution.

M. E. Germain, in a recent article on crime, says that some of the responsibility for those epidemics of crime which we know so well belongs to those journalists who, passing over the real tragedies of life, those of innocence and misfortune, shed the lurid glow of publicity around the criminal. Reading the elaborate accounts of murders and murderers our newspapers give, others whose criminal impulses might have remained dormant for ever, are impelled to imitate the deeds they read of.

"Every man his own doctor" is not a safe rule, and the idea that a man is either a fool or a physician by the time he has reached forty may be carried to untoward conclusions by the fools. Recently a gentleman, aged forty, saw an account of the treatment of locomotor ataxy by suspension, and having, or thinking he had, some of the symptoms of the disease, he bought an apparatus and attempted to treat himself. His footman assisted at and supervised the experiments. The first seven suspensions had no particular effect; during the eighth he lost the power of speech and hearing, and by degrees became totally paralysed, death resulting within twenty-four hours.

